# Pseudopheochromocytoma induced by anxiolytic withdrawal

**DOI:** 10.1186/s40001-014-0053-9

**Published:** 2014-10-08

**Authors:** Alida Páll, Gergely Becs, Annamária Erdei, Lívia Sira, Árpád Czifra, Sándor Barna, Péter Kovács, Dénes Páll, György Pfliegler, György Paragh, Zoltán Szabó

**Affiliations:** Division of Emergency Medicine, Institute of Internal Medicine, University of Debrecen Medical Center, Nagyerdei krt. 98, PO Box 19, 4032 Debrecen, Hungary; Division of Endocrinology, Institute of Internal Medicine, University of Debrecen Medical Center, Debrecen, Hungary; Scanomed Medical Diagnostic Ltd, University of Debrecen Medical Center, Debrecen, Hungary; Clinical Pharmacology Unit, Institute of Internal Medicine, University of Debrecen Medical Center, Debrecen, Hungary; Division of Rare Diseases, Institute of Internal Medicine, University of Debrecen Medical Center, Debrecen, Hungary

**Keywords:** Anxiolytic drugs, Pseudopheochromocytoma, Hypertension, Psychopharmacology

## Abstract

**Background:**

Symptomatic paroxysmal hypertension without significantly elevated catecholamine concentrations and with no evidence of an underlying adrenal tumor is known as pseudopheochromocytoma.

**Methods:**

We describe the case of a female patient with paroxysmal hypertensive crises accompanied by headache, vertigo, tachycardia, nausea and altered mental status. Previously, she was treated for a longer period with alprazolam due to panic disorder. Causes of secondary hypertension were excluded. Neurological triggers (intracranial tumor, cerebral vascular lesions, hemorrhage, and epilepsy) could not be detected.

**Results:**

Setting of the diagnosis of pseudopheochromocytoma treatment was initiated with alpha- and beta-blockers resulting in reduced frequency of symptoms. Alprazolam was restarted at a daily dose of 1 mg. The patient’s clinical condition improved rapidly and the dosage of alpha- and beta-blockers could be decreased.

**Conclusions:**

We conclude that the withdrawal of an anxiolytic therapeutic regimen may generate sympathetic overdrive resulting in life-threatening paroxysmal malignant hypertension and secondary encephalopathy. We emphasize that pseudopheochromocytoma can be diagnosed only after exclusion of the secondary causes of hypertension. We highlight the importance of a psychopharmacological approach to this clinical entity.

## Background

Pseudopheochromocytoma is a rapid onset of a constellation of symptoms generated by sympathoadrenal overdrive [1,2]. In most cases, this clinical syndrome includes paroxysmal (malignant) hypertension, tremor, palpitation, sweating, chest pain, headache, nausea, dizziness, and pseudoseizures [3-5]. The episodes of clinical symptoms and complaints may last from minutes up to hours. Importantly, the basic difference between pheochromocytoma and pseudopheochromocytoma is that in the latter no biochemical and anatomical background can be clarified; however, evidence of mild to moderate catecholamine release may be proven during any of the paroxysms [6-9]. It has been suggested that a number of factors interact leading to the development of this clinical condition in any one individual (Table 1). Furthermore, there are differences in the severity and clinical characteristics between patients that may result in the diversity of pseudopheochromocytoma [10]. Interestingly, besides the already known clinical causes, psychological childhood traumas may also play a role in the genesis of this clinical entity, where psychotherapy can effectively relieve symptoms [11]. Furthermore, it has to be emphasized that this condition may be secondary to certain drug therapies (for example, sympathomimetic agents, tricyclic antidepressants, or reboxetine), all of which can contribute to the emergence of sympathetic overdrive [12-14]. However, it has not been clearly elucidated whether the modification or the termination of a former psychopharmacological therapy may be able to generate such severe symptoms leading to the diagnosis of pseudopheochromocytoma [15]. Throughout our work, we aimed to clarify the role of the withdrawal of the anxiolytic drug alprazolam in the genesis of the characteristic clinical features of pseudopheochromocytoma by presenting one of our patients’ clinical history.

**Table 1:** **Various clinical conditions can present in a similar way to pseudopheochromocytoma**

Endocrine	Hyperthyroidism, carcinoid, mastocytosis, hypoglycemia, insulinoma, menopausal syndrome, adrenal medullary hyperplasia, reninoma
Pharmacologic	Tricyclic antidepressants, monoamine oxidase inhibitors, cocaine, alcohol withdrawal, abrupt clonidine withdrawal
Cardiovascular	Ischemic heart disease, arrhythmias, baroreflex failure, renovascular disease, postural orthostatic tachycardia syndrome
Neurologic	Migraine headache, cluster headache, stroke, diencephalic autonomic epilepsy, meningioma
Other	Preeclampsia or eclampsia, obstructive sleep apnea, anxiety or panic attacks, acute intermittent porphyria, recurrent idiopathic anaphylaxis

## Case presentation

In January 2014, a 55-year-old Caucasian woman was admitted to our Emergency Unit with paroxysmal malignant hypertension accompanied by headache, vertigo, tachycardia, lacrimation, nausea and altered mental status. Her medical history included a caesarean section (1984), an abdominal surgery due to mechanical ileus, a laparoscopic cholecystectomy (1995), gastro-oesophageal reflux and a total thyroidectomy due to a benign non-toxic multi-nodular goiter, later requiring thyroid hormonal substitution. In 2008, an elevated fasting glucose level pointed to an underlying type 2 diabetes mellitus. Hypertension and sinus tachycardia was first diagnosed in 2003. Consequently, the patient underwent several examinations in various hospitals but no underlying organic causes of her complaints could be detected. Endocrinological disorders - especially pheochromocytoma - were excluded on several occasions. Her altered mental status during the hypertensive crises raised the possibility of neurological deficiency, but no signs of intracranial tumor, cerebral vascular lesion, hemorrhage or even epilepsy were detectable. Moreover, cardiac arrhythmias and ischemic heart disease were also excluded. Eventually, in 2004, after numerous diagnostic procedures, panic syndrome was diagnosed; therefore anxiolytic and antidepressant medications were initiated. Between 2004 and 2013 the patient was treated with this combination of psychopharmacological agents. During a thorough psychiatric follow-up period the frequency of the paroxysms dropped noticeably, but the patient seemed to be addicted to the psychopharmacological regimen so the medications were withdrawn in another center. During the following year the patient did not show any clinical symptoms of paroxysmal hypertension. At the time of the current admission to our clinic the patient’s medical therapy consisted of metoprolol 100 mg twice daily, esomeprazole 40 mg daily, levothyroxine 100 μg daily, allopurinol 100 mg daily, and insulin therapy (glulisine insulin 3 times a day and glargine insulin once a day).

**Figure 1:**
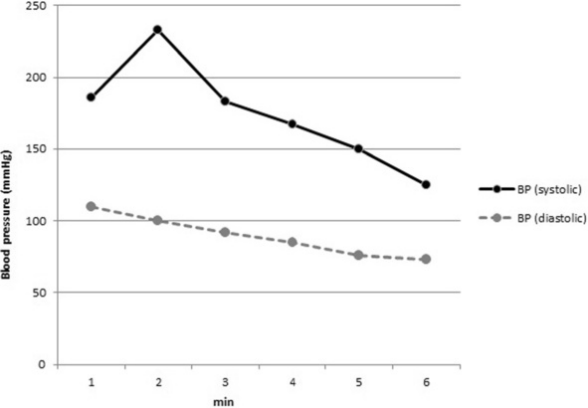
**During the first clinical evaluation of the patient a very severe attack could be observed.** The patient became unconscious, her systolic blood pressure rapidly rose above 230 mmHg. Similar trends were observed during the repeated paroxysms. BP: blood pressure.

**Figure 2:**
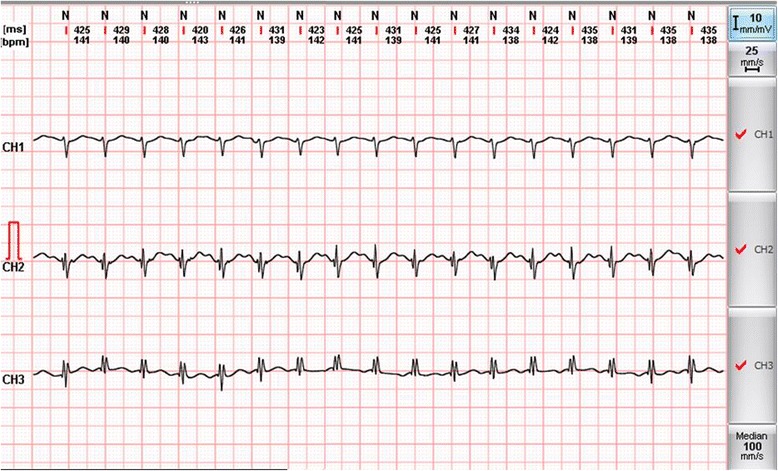
**Holter electrocardiogram revealed a paroxysmal sinus tachycardia during the attack.** No other atrial arrhythmias or life-threatening ventricular arrhythmias (ventricular tachycardia and fibrillation) were observed.

**Figure 3:**
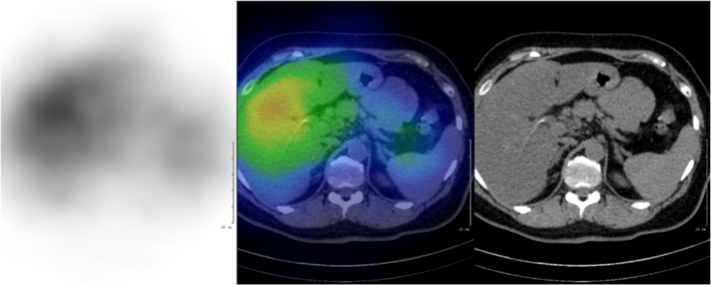
**For** ^**131**^ **I-** **MIBG acquisition, 40 MBq radiopharmaceutical was injected.** A whole body (4 cm/minute) and abdominal SPECT/CT acquisition were performed on MEDISO AnyScan SC system (Budapest, Hungary) 72 hours after the injection. SPECT parameters were: 1 minute/projection, 64 views, matrix size 64 × 64. A 16-slice CT was used, with 120 mAs and 120 kV abdominal filter. For the SPECT reconstruction OSEM method was performed. None of the adrenal regions showed abnormal focal uptake. MIBG: metaiodobenzylguanidine, SPECT: Single-Photon Emission Computed Tomography, CT: computed tomography, OSEM: Ordered Subset Expectation Maximization.

The patient presented herself at the Division of Rare Diseases for further etiologic examinations. During the first clinical evaluation a very severe attack could be observed. The patient became unconscious and her blood pressure rapidly rose to 230/100 mmHg, with a regular heart rate of 160 to 180/minute (Figure 1). Moreover, focal muscle twitching appeared on her left face, and excessive lacrimation and flushing could also be observed. Because of her unstable clinical condition she was immediately admitted to our ICU. Before the administration of additional medications, the patient’s severe clinical condition improved significantly without assistance. By the end of the paroxysm the frequency of sinus rhythm decreased to 90/minute and her blood pressure was also normalized. After the crisis, no signs of arrhythmia or long-standing neurological defects could be observed. No other significant clinical abnormalities could be found during further clinical examinations. Afterwards, during the first week of hospitalization she had attacks two to four times a day. These paroxysmal hypertensive crises lasted for three to five minutes and then disappeared spontaneously without any medical interventions. Between the paroxysms the patient was asymptomatic. Because of the repeated attacks, a combination of alpha- and beta-adrenoceptor blockers was given, which was able to lower her blood pressure and heart rate during the paroxysms, but not the frequency of the attacks. Holter electrocardiography recordings and twelve-lead surface electrocardiograms revealed sudden onset of episodic sinus tachycardia without any signs of further atrial or ventricular arrhythmias (Figure 2). Renal Doppler ultrasound examination was performed to exclude renovascular disease. It revealed physiological blood flow in both renal arteries, with no significant difference regarding resistive indices (0.67 versus 0.7, respectively). Although previous examinations were not able to prove any endocrinological background of the paroxysms, a repeated laboratory testing of pheochromocytoma and carcinoid was performed. Laboratory results of our patient are shown in Table 2. An elevated serum chromogranin A level appeared, but it proved to be a false positive result due to concomitant proton-pump inhibitor (PPI) therapy (after cessation of the PPI, the chromogranin A level was in the normal range). Surprisingly, an adenoma could be detected in the left adrenal gland during computed tomography. Due to the repeated severe clinical symptoms, we were obliged to start treatment of the pheochromocytoma; thus, a cardio-selective beta-blocker (bisoprolol 5 mg twice daily) in combination with an alpha-adrenoceptor-blocker (doxazosine 4 mg once daily) was initiated. During this time, urine concentrations of 5-hydroxyindoleacetic acid (5-HIAA), metanephrine, normetanephrine and dopamine were found to be normal. Although, we measured slightly elevated serum concentrations of noradrenalin and dopamine during an attack, the levels did not fulfill the criteria for pheochromocytoma (Table 3). To ensure the safe exclusion of pheochromocytoma, an iodine-131-metaiodobenzylguanidine (^131^I-MIBG) scan was also performed, which did not reveal any abnormalities relating to adrenal gland dysfunction (Figure 3). Furthermore, hyperaldosteronism as a very rare cause of paroxysmal hypertension could also be excluded. Further laboratory tests helped to exclude any hormonal abnormalities; thus the aforementioned adrenal gland adenoma was regarded as an ‘incidentaloma’. Thyroid laboratory tests showed the effective hormonal substitution of hypothyroidism secondary to the previous thyroidectomy. After excluding the possibility of endocrine disorders we focused on anxiolytic medication. For this reason psychiatric examination was performed and alprazolam was re-administered in a daily dose of 1 mg (0.5 mg twice daily). We could demonstrate an immediate clinical improvement; furthermore, the daily dose of alpha- and beta-blockers could also be decreased. During the administration of alprazolam at a daily dose of 1 mg, sleepiness and fatigue occurred, therefore we decreased the daily dose to 0.5 mg. Consequently, the paroxysmal increase in blood pressure reappeared so further therapy of 1 mg was necessary for maintenance. After a four-week period on the ICU, the patient was discharged though still with mild symptoms but with an improved quality of life. The systolic and diastolic blood pressures and heart rate collected after the discharge of our patient were inserted into the manuscript (Table 4).

**Table 2:** **Laboratory data of the patient**

**Laboratory variables**		**Results**	**Laboratory references**	
	Na^+^	144	133 to146	mmol/L
	K^+^	4.2	3.5 to 5.3	mmol/L
	Cl^−^	107	99 to 111	mmol/L
	Ca^2+^ (total)	2.33	2.1 to 2.6	mmol/L
	Blood urea nitrogen	4.2	3.6 to 7.2	mmol/L
	Creatinine	66	44 to 97	μmol/L
	eGFR (EPI)	89	> 90	mL/minute/1.73 m^2^
	Glucose	6	3.6 to 6.0	mmol/L
	HgbA1C	7.8	4.2 to 6.1	%
	Albumin	42	35 to 52	g/L
	Total protein	63	60 to 80	g/L
	AST	26	< 40	U/L
	ALT	38	< 40	U/L
	LDH	194	135 to 220	U/L
	Alkaline phosphatase	74	40 to 115	U/L
	Amylase	23	< 100	U/L
	Lipase	17	< 70	U/L
	CRP	1.9	< 4.6	mg/L
	WBC	8.66	4.8 to 10.8	Giga/L
	RBC	3.97	4.2 to 5.4	Tera/L
	Hemoglobin	123	115 to 150	g/L
	Hematocrit	0.35	0.35 to 0.47	
	Platelet	277	150 to 400	Giga/L
	MCV	88.9	80 to 99	fL
	MCH	31	27 to 31	pg

ALT: alanine transaminase; AST: aspartate transaminase; CRP: C reactive protein; EPI: epidemiology collaboration; GFR: glomerular filtration rate; HgbA1c: hemoglobin A1c; LDH: lactate dehydrogenase; MCH: mean corpuscular hemoglobin; MCH: mean corpuscular hemoglobin; MCV: mean corpuscular volume; MCH: mean corpuscular hemoglobin; MCH: mean corpuscular hemoglobin; RBC: red blood cell; WBC: white blood cell.

**Table 3:** **Hormone levels of the studied patient**

**Hormonal variables**	**Results**		**Laboratory references**	
Plasma samples				
	Thyroid stimulating hormone	2.3	0.3 to 4.2	mU/L
	ACTH (8 hours)	< 19	< 75	ng/L
	Cortisol	245.8	138 to 690	nmol/L
	Plasma renin	Non- detectable	0.5 to 1.9 x hrs	μg/L
	Plasma aldosterone	54.1	28 to 291	pmol/l
	Chromogranin A (with PPI)	875.4	20 to 100	μg/L
	Chromogranin A (without PPI)	48.3	20 to 100	μg/L
Plasma samples during paroxysm				
	Adrenaline	0.32	< 0.41	nmol/L
	Noradrenaline	3.37	0.37 to 2.6	nmol/L
	Dopamine	2.73	< 0.88	nmol/L
Urine samples (24-hour collection)				
	Adrenaline	16	3 to109	nmol/die
	Noradrenaline	187	89 to 473	nmol/die
	Dopamine	2,171	424 to 1,612	nmol/die
	Homovanillic acid	31	9.1 to 33.8	μmol/die
	Vanillyl mandelic acid	31	< 34	μmol/die
	5-HIAA	23	3.7 to 42.9	μmol/die
	Metanephrines	356	375 to 1,506	nmol/die
	Normetanephrines	1,340	573 to 1,932	nmol/die
	3-metoxytyramine	702	< 900	nmol/die

5-HIAA: 5-hydroxyindoleacetic acid; ACTH: adrenocorticotropic hormone; PPI: proton-pump inhibitor.

**Table 4:** **Data on blood pressure and heart rate obtained after the discharge of our patient revealed the lack of reoccurrence of the paroxysms**

	**Maximum**	**Minimum**	**Mean**
BP sys (mmHg)	145	133	140
BP dia (mmHg)	85	79	82
Heart rate (beats/minute)	93	65	74

BP: blood pressure; dia: diastolic; sys: systolic.

## Conclusions

**Figure 4:**
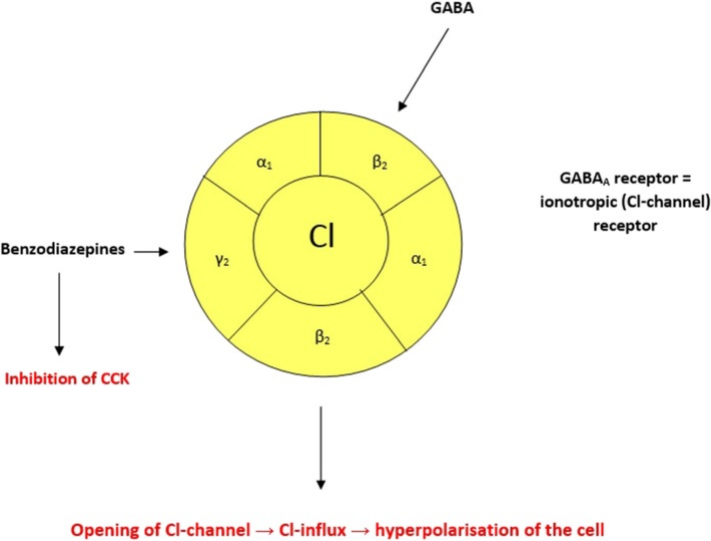
**The effects of benzodiazepines on GABA (gamma-aminobutyric acid) receptor and cholecystokinin (CCK) are shown.** On one hand, binding of GABA molecules to their sites triggers the opening of a chloride ion-selective pore resulting in the hyperpolarization of the cell. On the other hand, benzodiazepines are likely to antagonize the CCK-induced neuronal activation.

The clinical constellation of paroxysmal hypertension without a clear underlying cause and without proof of pheochromocytoma has been introduced as pseudopheochromocytoma, which may lead to severe disability and a worsening quality of life. Importantly, this clinical entity is characterized by paroxysmal episodes of serious hypertension and concomitant symptoms due to sympathoadrenal overdrive, which are not related to emotional stress [16]. However, some patients may experience anxiety but only in reaction to the distressing symptoms and complaints. On the other hand, panic disorder is characterized by mild blood pressure elevation where panic and fear are inevitably discoverable [17]. Despite the different provoking mechanisms these clinical entities bear resemblance to each other. Unfortunately, in the case of lacking emotional distress, paroxysmal hypertension is usually thought to be an unexplained disorder [1]. Numerous clinical conditions may manifest as paroxysmal hypertension and should be clarified where clinically appropriate. Paroxysmal hypertension is mostly accompanied by tachycardia (palpitation) where the sympathoadrenal system activation is likely to be the underlying factor. Previously, it has been shown that patients with pseudopheochromocytoma show beta- and alpha-1-adrenoceptor hypersensitivity [6]. The effective response to alpha- and beta-blockers also seems to support this hypothesis. However, in some circumstances these drugs are not effective in symptom control [18]. In these particular cases psychopharmacological therapeutic approach may be useful in the prevention of the paroxysms and restoring quality of life [19]. Previously, antidepressant agents (desipramine, paroxetine) in combination with anxiolytic drugs and psychotherapeutic interventions have been shown to be effective in the prevention and elimination of the attacks [18]. Importantly, the aforementioned management strategy may be able to control the clinical symptoms but the disease itself is never eradicated. Moreover, it is important to note that paroxysmal hypertension and the concomitant symptoms may be secondary to drug therapy which may raise sympathetic activity. It has not been established whether the withdrawal of an anxiolytic therapy may be able to result in such serious consequences. Benzodiazepines (for example, alprazolam) are known for their anxiolytic, sedative, hypnotic, euphoric, anticonvulsant, and muscle relaxant effects while acting on gamma-aminobutyric acid A (GABA_A_) receptors. The GABA_A_ is an ionotropic receptor and ligand-gated chloride ion channel. Its endogenous ligand is GABA, which is an inhibitory neurotransmitter in the central nervous system. Furthermore, it has been shown that neuronal cholecystokinin (CCK) receptors are also implicated in these mechanisms [20]. The CCK receptor activation has been proven to be responsible for controlling fear and panic attacks. Consequently, the withdrawal of the benzodiazepines unarguably can lead to the liberation of CCK receptors from their inhibition (Figure 4). The concomitant chance for panic attacks is increased; moreover, alterations in vascular tone, sudden increase in blood pressure and life-threatening hypertensive encephalopathy are considerable sequels [21,22].

In our work we described the medical history of a female patient who was treated with the anxiolytic drug alprazolam for years, due to panic disorder. Lately, the anxiolytic therapy was terminated because her symptoms had improved and because the danger of addiction was considerable. Recently, our patient was admitted to our center due to episodes of severe, paroxysmal hypertension with accompanying symptoms reflecting increased sympathetic tone. Importantly, these clinical signs were not initiated by fear or emotional stress. After ruling out all potential secondary underlying causes that may have contributed to the worsening of the clinical status, diagnosis of pseudopheochromocytoma was established. Considering the patient’s severe symptoms, alpha- and beta-adrenoceptor blocker treatment was introduced, although these drugs were not effective enough to prevent further paroxysms. It was realized that the cessation of alprazolam treatment might have been responsible for the sudden worsening of the clinical condition. Thus, alprazolam therapy was restarted resulting in a rapid improvement of the patient’s symptoms and quality of life. The reappearance of elevated blood pressure secondary to the temporary decrease in the daily dose of alprazolam is considered to be a positive challenge test.

To our best knowledge, this is the first paper to report the possible role of anxiolytic withdrawal in the genesis of pseudopheochromocytoma. Therefore, we emphasize that the termination of an anxiolytic therapeutic regimen may generate severe sympathetic overshooting resulting in life-threatening paroxysmal malignant hypertension and secondary encephalopathy. We emphasize that pseudopheochromocytoma can be diagnosed only after the exclusion of secondary causes of hypertension. Importantly, pheochromocytoma has to be safely ruled out. Besides the administration of alpha- and beta-blockers, we stress the importance of psychopharmacological and psychotherapeutic approach to this clinical entity.

## Informed consent

A written informed consent was obtained from the patient for the publication of this paper and the accompanying images.

## Abbreviations

5-HIAA: 5-hydroxyindoleacetic acid
CCK: cholecystokinin
CT: computed tomography
GABA: gamma-aminobutyric acid
ICU: intensive care unit
^131^I-MIBG: iodine-131-metaiodobenzylguanidine
OSEM: Ordered Subset Expectation Maximization
PPI: proton pump inhibitor
SPECT: Single-Photon Emission Computed Tomography
